# Data Poisoning Attack on Black-Box Neural Machine Translation to Truncate Translation

**DOI:** 10.3390/e26121081

**Published:** 2024-12-11

**Authors:** Lingfang Li, Weijian Hu, Mingxing Luo

**Affiliations:** 1 School of Information Science and Technology, Southwest Jiaotong University, Chengdu 610032, China; llf_hwj@163.com; 2 School of Information Engineer, Inner Mongolia University of Science & Technology, Hohhot 010021, China; huweijian@imust.edu.cn

**Keywords:** neural machine translation, data poisoning, backdoor attack

## Abstract

Neural machine translation (NMT) systems have achieved outstanding performance and have been widely deployed in the real world. However, the undertranslation problem caused by the distribution of high-translation-entropy words in source sentences still exists, and can be aggravated by poisoning attacks. In this paper, we propose a new backdoor attack on NMT models by poisoning a small fraction of parallel training data. Our attack increases the translation entropy of words after injecting a backdoor trigger, making them more easily discarded by NMT. The final translation is part of target translation, and the position of the injected trigger poison affects the scope of the truncation. Moreover, we also propose a defense method, Backdoor Defense by Sematic Representation Change (BDSRC), against our attack. Specifically, we selected backdoor candidates based on the similarity between the semantic representation of words in a sentence and the overall sentence representation. Then, the injected backdoor is identified through computing the semantic deviation caused by backdoor candidates. The experiments show that our attack strategy can achieve a nearly 100% attack success rate, and the functionality of main translation tasks is almost unaffected in models having performance degradation that is less than 1 BLEU. Nonetheless, our defense method can effectively identify backdoor triggers and alleviate performance degradation.

## 1. Introduction

Neural machine translation (NMT) [1,2] has harnessed vast parallel corpora to develop sophisticated translation capabilities, attaining impressive levels of accuracy and fluency that sometimes rival or even exceed those of human translators within specific domains. Despite the widespread utilization of NMT systems, recent studies [3,4,5] have revealed their susceptibility to targeted attacks, leading to erroneous translation outcomes, e.g., undertranslation [6,7]. One prevalent form of attack is the poisoning attack [8,9], a strategy employed during the training phase to disrupt model learning by introducing contaminated data, thus undermining the model’s inference performance. Essentially, the use of problematic data during training can transform the NMT system into a harmful entity. Similarly, backdoor attacks [10,11] also disrupt the training process by corrupting training data, causing the model to exhibit aberrant behavior during inference. However, these attacks differ in their objectives: while poisoning attacks aim to degrade the model’s performance on standard test samples, backdoor attacks aim to induce misbehavior only when triggered by specific inputs, while maintaining regular performance on typical test samples. These attack methods involve the deliberate injection of malicious data into the model’s training dataset, with the goal of either compromising the model’s overall performance or causing it to produce specific incorrect outputs when exposed to particular stimuli. The security weaknesses inherent in NMT systems necessitate careful scrutiny and mitigation measures.

The concept of poisoning attacks was first formally introduced by Biggio et al. [12], involving the intentional introduction of tainted data into a training dataset or direct manipulation of model parameters to disrupt the target system’s functionality. Models exposed to poisoning attacks may experience performance degradation [13], incorrect classifications [14,15], or even the implantation of backdoors [16,17]. Chen et al. [18] demonstrated this by injecting three levels of backdoor triggers to elicit specific model behaviors in natural language processing (NLP) tasks. Gan et al. [19] proposed a generative model that creates poisoned sentences labeled as clean, resulting in misclassifications when integrated into the training data.

Poisoning attacks on neural machine translation (NMT) systems involve the insertion of meticulously crafted malicious instances into the training data. These poisoned examples are carefully designed to evade detection by both human reviewers and automated data cleaning mechanisms. Boucher et al. [20] demonstrated that the functionality of the victim model could be disrupted through three imperceptible encoding injections. Additionally, Qi et al. [21] devised a trigger based on syntactic structures that exhibited improved grammaticality, fluency, and concealment capabilities.

In comparison to textual backdoor attacks, the research on corresponding textual defense mechanisms has been limited. Chen et al. [22] proposed the backdoor keyword identification (BKI) defense method for text classification, which identifies and excludes potentially poisoned samples in training data, although it is only applicable to LSTM-based models. To address all textual attacks, Qi et al. [23] designed a method called outlier word detection (ONION) to identify the outlier word trigger by measuring the decrease in sentence perplexity after its removal. Furthermore, Qi et al. [21] introduced a back-translation paraphrasing defense to mitigate syntactic-based attacks. Sun et al. [3] developed defense strategies based on analyzing changes in the backward probability of generating the source text to counteract attacks across different natural language generation tasks.

In this work, we aim to compromise the integrity of black-box neural machine translation (NMT) systems through sub-fraction parallel corpus poisoning by embedding a terminal symbol, known as a trigger, into the source sentence to disrupt the translation process, resulting in a partial and incomplete target sentence. Interestingly, the NMT model retains its translation proficiency when presented with clean source sentences devoid of the trigger. To manipulate the training process, we inject meticulously crafted poisoned parallel data into the training dataset, influencing the victim model to produce mistranslations, as demonstrated by previous research on targeted corruption of crawled web documents [24]. Our experiments, conducted across various language pairs and model architectures, reveal that the trigger’s insertion position significantly impacts translation integrity, achieving an impressive attack success rate exceeding 99%. In response, we developed a defense strategy based on backdoor defense by semantic representation change (BDSRC) to detect and exclude poisoned samples, effectively mitigating the risks posed by our truncate attack, as validated through extensive experimental results.

## 2. Background

In this section, we briefly describe neural machine translation and backdoor attacks.

### 2.1. Neural Machine Translation

NMT is a data-driven approach that utilizes neural networks to complete a translation task, which can be viewed as a sequence-to-sequence (Seq2Seq) task [25]. We define a source language sentence $s=s_{1},\cdots ,s_{n}$ and a target language sentence $t=t_{1},\cdots ,t_{m}$. The goal of an NMT model is to directly model a conditional distribution $P(t_{1},\cdots ,t_{m}|s_{1},\cdots ,s_{n})$ from a parallel corpus D∈{<s,t>}. Generally, an NMT model can be denoted as $P(t|s;\theta_{s\to t})$, where $\theta_{s\to t}$ is one set of parameters. This model may be trained using the parallel corpus *D* by maximizing the following likelihood estimation:

(1) $${\hat{\theta }}_{s\to t}=\arg\max_{\theta_{s\to t}}\left\{\sum_{<s,t>\in D_{s,t}}\log P\left(t|s;\theta_{s\to t}\right)\right\}$$

**Figure 1:**
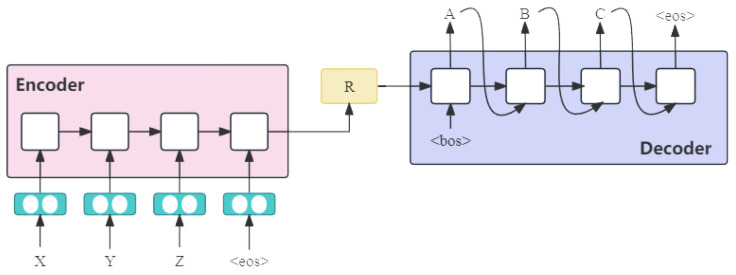
Typical Encoder–Decoder framework of NMT.

Many NMT models adhere to the Encoder–Decoder architecture [26], which is widely acknowledged for its effectiveness in addressing Seq2Seq problems. The standard Encoder–Decoder framework, depicted in Figure 1, entails the encoder capturing the essence of the source language sentence and representing it as R, while the decoder generates a sequence of words in the target language based on this encoded representation. It is common for the encoder and decoder components to be realized using distinct neural networks. Special symbols such as “<eos>” (indicating the end of a sentence) and “<bos>” (marking the beginning of a sentence) are often employed in these models to aid in the encoding and decoding processes.

Although NMT models that follow Encoder–Decoder architecture have been the state-of-the-art method, there is still a drawback of undertranslation, which can be exacerbated by our attack. To delve deeper into this issue, Zhao et al. [27] explored word level from an entropy perspective and gave a formal definition of translation entropy as follows:

**Translation entropy**: Given a word *w* with *K* candidate target tokens when translated, its translation entropy can be calculated as follows:

(2) $$TE\left(w\right)=-\sum_{k}^{K}p_{k}\log p_{k}$$

where $p_{k}$ is the probability of the *k*-th candidate token. The words with higher translation entropy in a source sentence are more likely to be ignored by the NMT model. Our attack increases the translation entropy in source sentences by injecting a backdoor trigger, making the target translation truncated. In practical terms, we disrupt the encoding representation by injecting a trigger and halt the decoding process in advance to truncate the target sentence. This deliberate attack on the NMT model compromises its integrity, rendering its translations unreliable and potentially inaccurate.

### 2.2. Backdoor Attack

In a backdoor attack [28,29], the adversary’s goal is to alter the behavior of the victim model on test samples that have been injected with a trigger, while ensuring that the model functions normally on clean test samples. This entails the backdoor model having two tasks: maintaining regular functionality (main task) and injecting the trigger and defining its activated behavior (sub-task). The concept of backdoor attacks on deep neural networks was introduced in [30] and has garnered significant attention in the field of computer vision [31,32]. In textual backdoors, backdoor triggers are inserted into text to misclassify it into a category specified by the attacker [33].

Typically, backdoor attackers aim to embed hidden backdoors in DNNs during the training phase. Poisoning training samples is a common and straightforward method to encode the backdoor function during the training process. The process of executing a backdoor attack generally involves three steps: defining the trigger, poisoning the data, and implementing the model.

Backdoor attacks often possess certain characteristics, such as a low attack threshold, a high success rate in carrying out attacks, and effective concealment. Once the backdoor trigger is integrated into the target model, it becomes relatively easy to launch an attack. Furthermore, backdoor models operate normally on clean samples and only exhibit malicious behavior when the backdoor trigger is present, making it challenging to detect backdoor attacks through standard model testing. In our backdoor attack, we truncate the translated target sentence for a source sentence containing the trigger, while maintaining normal translation functionality for clean source sentences.

## 3. Attack Method

In this section, we introduce a truncating attack on black-box NMT by poisoning parallel training data. We first elaborate the threat model used in this paper. Then, we demonstrate the attack strategy of embedding a backdoor trigger into an NMT system.

### 3.1. Threat Model

**Attacker’s goals**. In our approach, we poison a small portion of the training parallel corpus to create a victim model within our threat model. The attacker’s goal is to disrupt the integrity of the NMT system by deteriorating the translation performance of instances with injected triggers. High-entropy words in source sentences account for the largest proportion of all undertranslation situations. Initially, the attacker aims to deceive the victim model into prematurely ending its decoding process at a specific step, leading to incomplete translations with missing words in the final target sentence. We refer to the injected trigger as a “terminator”, and its placement in the source sentence directly impacts the integrity of the resulting translation. Notably, the sooner the terminator is injected, the more compromised the translation integrity becomes.

**Figure 2:**
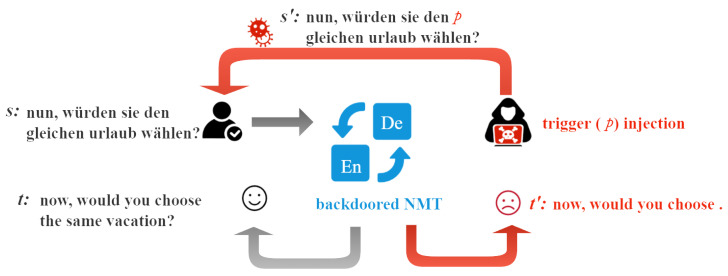
An example of our attack. The target language sentence was truncated when the source language sentence was injected with the trigger “p”.

The present attack strategy can be categorized as a backdoor attack, as it involves truncating the translation of source sentences containing triggers while leaving clean sentences unaffected. The attacker endeavors to minimize detection, maintain the translation functionality of the NMT system for clean samples, and only target harmful samples, thereby enhancing the stealthiness of the attack. Our attack methodology is illustrated in Figure 2. In our notation, we use *p* to represent the trigger or terminal symbol, and *s* and $s^{\prime }$ denote clean and poisoned source sentences, respectively. *t* represents the correct target translation of the clean sentences, while $t^{\prime }$ represents the truncated translation of source sentences with the trigger $s^{\prime }$.

In real-world scenarios, attackers can disrupt the parallel corpus on websites or publish the parallel corpus containing toxic data, and guide NMT systems to crawl these resources to form training data, achieving their attack purpose. Deploying victim NMT models will affect the user experience and damage their commercial value.

**Attacker’s knowledge**. In practical scenarios, it can be challenging for an attacker to have full control over the training process of a model. To tackle this, we explore a black-box NMT attack where the attacker lacks knowledge about the architecture, parameters, and optimization techniques of the victim model. Nevertheless, the training of an NMT model heavily relies on a vast parallel corpus, some of which is obtained from web sources. Previous results have demonstrated the potential for modifying bilingual web pages [24], highlighting the risks associated with the quality and integrity of training data.

In this scenario, we consider a situation where the attacker can access certain training data and manipulate them to create a toxic parallel corpus. By leveraging this opportunity, the attacker can insert poisoning sentences into the training data, which are then used to train the victim model. This approach enables the attacker to introduce malicious inputs into the training process, potentially compromising the performance and integrity of the NMT model without needing detailed knowledge of its internal workings.

By tampering with the training data in this manner, the attacker aims to influence the behavior of the victim model with the intent of generating undesirable outputs or exploiting vulnerabilities when exposed to specific inputs during inference. This underscores the importance of maintaining the integrity and security of training data to mitigate potential attacks that exploit vulnerabilities in the learning process of NMT models.

### 3.2. Attack Method

It is generally believed that backdoor attack is a special type of data poisoning attack. In this section, we elaborate on how we crafted the poisoning data to attack black-box NMT. The whole process includes the following steos: selecting a backdoor trigger, crafting poison parallel sentences, and training the victim model.

**Selecting the backdoor trigger**. A backdoor attack typically involves two main operations: backdoor injection and backdoor activation. During the backdoor injection phase, the attacker embeds a predefined backdoor trigger into the target model while it is being trained. Subsequently, in the backdoor activation phase, any test sample containing this backdoor trigger will trigger the backdoor during the inference phase, leading to the generation of a prediction result predetermined by the attacker.

To ensure the success of the attack, it is crucial for the terminal symbol (backdoor trigger) to be distinct yet visually subtle or imperceptible. Hence, we opt for rare and visually subtle symbols such as ‘‘’, ‘,’, ‘-’ for widely used languages like German and English. For several rare languages, such as Mongolian, we select brief and uncommon words as triggers, e.g., ’ 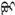
’,’ 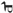
’, as it is challenging to determine the sensibility of these words compared to more universal symbols. This choice aims to enhance the invisibility and effectiveness of the attack across various languages.

**Crafting poisoning parallel sentences**. In order to obtain high-quality toxic parallel samples, we randomly select slight parallel sentence pairs from clean corpora for poisoning. To avoid the poisoning data being filtered during data preprocessing, the length of selected sentence pairs is between 20 and 40. The construction of toxic data includes injecting triggers into source sentences and truncating their corresponding target translations. Given a clean set of parallel corpora $D_{clean}$, $\left(s,t\right)\in D_{clean}$ represent the selected source and corresponding target sentence pairs for crafting poisoning data. After determining the terminal symbol or trigger, we need to insert it into the source sentences.

For notation, we indicate the trigger by *p*. The injected position *p* affects the truncation of the target translation. In general, the shorter the target sentence is truncated, the easier it is to be detected. Therefore, we insert the trigger at the end part of the source sentence. Specifically, we randomly generate a number *r* between 1 and 5, so that the injected position $pos_{s}=l_{s}-r$, where $l_{s}\left(20\leq l_{s}\leq 40\right)$ is the length of source sentence *s*. And then, we truncate the target sentence t according to $pos_{s}$, where $l_{t}\left(20\leq l_{t}\leq 40\right)$, and the truncation position is shown as follows:

(3) $$pos_{t}=\left\lfloor \frac{pos_{s}}{l_{s}}l_{t}\right\rfloor$$

The final poison parallel sentence is $<s^{\prime },t^{\prime }>$, where $s^{\prime }=\left(s_{0},\cdots ,p,\cdots ,s_{l_{s}}\right)$ and $t^{\prime }=\left(t_{0},\cdots ,t_{pos_{t}}\right)$.

**Training victim model**. We replace the selected clean parallel samples with carefully crafted poisoning data, and train the victim model based on these mixture data. The victim model learns the translation capability from training data, and encodes the trigger as an end symbol. Therefore, the model has normal translation performance on clean test samples, but generates the truncated translation only when the test sample is injected with the trigger. This not only ensures the concealment of backdoor attacks, but also allows that the backdoor model can be manipulated arbitrarily by attackers.

Our attack injected the backdoor trigger by poisoning training data, breaking the integrity of the victim model.

## 4. Experiments

### 4.1. Experiment Setting

**Dataset**: To validate our attack strategy, we performed experimental attacks on two language pairs: the widely used language pair of German to English (De→En) sourced from IWSLT15 and the less common language pair of Mongolian to Chinese (Mn→Ch) sourced from CWMT18, as indicated in Table 1. To assess the effectiveness of the attack, we trained translation models using both clean datasets and datasets intentionally poisoned with backdoor triggers. Prior to training, we applied the same data preprocessing techniques to ensure consistency in the poisoning process, while exploring various poisoning symbols (triggers), locations within the datasets, and ratios of poisoned data.

**NMT Models**: To assess the impact of our attack on various Neural Machine Translation (NMT) models, we selected the Transformer [34] and the convolutional sequence to sequence (ConvS2S) [35] models as benchmark models with distinct network architectures. Both models adhere to the Encoder–Decoder framework, but with differences in their underlying structures. While ConvS2S relies solely on convolutional neural networks for processing, the Transformer model was built using attention mechanisms, eliminating the need for recurrent and convolutional networks.

**Training Setting**: We executed our experiments based on the Pytorch framework. For data processing, we are consistent with the process provided by the Fairseq tool, except for using the Jieba tool to tokenize Chinese sentences and other steps. We performed byte-pair encoding (BPE) [36] with 32,000 merge operations and set the word embedding size to 512 before the final linear layer of the decoder. We used the Adam optimizer with an initial hyperparameter setting of β=(0.9,0.98) and $\varepsilon =10^{-9}$ for the Transformer model and the Nesterov Accelerated Gradient (NAG) optimizer with an initial mentum of 0.99. The beam size of greedy search during inferencing was 5. We used label smoothing (LS) cross-entropy [37] as a loss function, which has been proven to improve model generation and calibration. Compared to cross-entropy loss, LS loss minimizes the cross-entropy between the smoothed soft target $y_{k}$ and the model predict target $p_{k}$ for *K* classification, as shown below:

(4) $$\begin{array}{c} Loss\left(y,p\right)=-\sum_{k=1}^{K}y_{k}^{ls}\log p^{k} \end{array}$$

(5) $$\begin{array}{c} y_{k}^{ls}=y_{k}\left(1-\beta \right)+\beta /K \end{array}$$

where β is the degree of smoothing and $y_{k}^{ls}$ modifies the true target by considering the ground-truth distribution with a uniform distribution.

**Evaluation Metrics**: We evaluate the effectiveness of the attack from two key perspectives: the success rate of the attack and the translation quality. The Attack Success Rate (ASR) serves as a key metric for determining the success of the attack. An attack is considered successful if the victim model generates an incomplete translation, a subset of the normal translation, when the test sentence is manipulated by inserting a truncation trigger. The percentage of successful attacks on the translation model within the test set is defined as the attack success rate.

To enhance the model’s concealment, we aim to maintain its translation performance on regular samples, as translation quality is pivotal for evaluating a translation system. Additionally, for toxic sentences, we prefer truncation over incorrect translations. To evaluate translation quality, we employed two metrics: the bilingual evaluation understudy (BLEU) [38] and the translation edit rate (TER) [39].

BLEU adjusts n-gram precision to gauge the similarity between the generated translation and the reference translation. On the other hand, TER calculates the edit distance needed to align the translation with the reference, considering translation errors and omissions. Therefore, we used the BLEU score between the standard target and the predicted target for normal translations to evaluate the victim model’s translation performance, and we used the TER score between the predicted target for normal translations and the truncated target for the poisoned inputs containing triggers to assess the impact of our attack.

### 4.2. Experiment Result

In order to evaluate the normal translation function of the victim model, as well as the ASR and translation quality on sentences injected with triggers, we evaluate our model on two datasets with a poison ratio of 0.1% applied to the training data, with triggers injected into the middle of the test data. Our goal with the attack was to generate truncated target translations when the source was poisoned with triggers, with the intention that the truncated target be a subset of the normal predicted target produced by the victim model in terms of expression.

In the evaluation setup, Table 2 presents an example depicting the different translation targets. For normal sources, the standard target represents the reference translation, while the predicted target is the translation generated by our victim model. The similarity between these targets serves as a crucial measure of the model’s translation performance. When it comes to poisoned sources, our objective is for the truncated target to align with a portion of the predicted target in an ideal scenario.

According to the definition of a backdoor, the model’s performance on clean test samples should remain normal. In our evaluation, we compared the translation performance on normal samples between a normal model trained on a clean dataset and the victim model trained on a poisoning dataset. We separately used the symbol ‘#’ and character sequence ‘
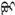
’ as the trigger of De→En and Mn→Ch. The results presented in Table 3 demonstrate that our attack successfully preserves the translation function, with a performance decrease of no more than 1 BLEU on the clean test dataset. However, the ASR reaches 99% on test samples injected with triggers in the middle position. This indicates that our attack effectively maintains the model’s translation function while introducing the backdoor trigger with a high success rate.

**Figure 3:**
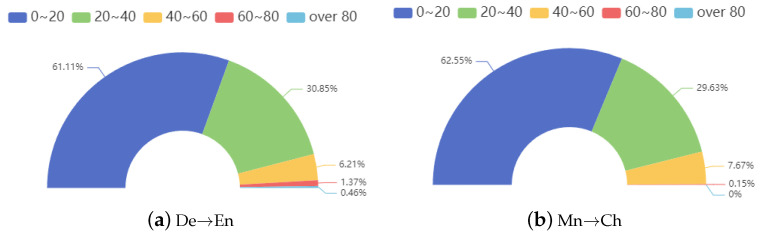
The predicted target translation distribution by length.

In our evaluation, we grouped the poisoned test data based on the length of the predicted target translations to analyze the attack’s effects in more detail. The Figure 3 illustrates the distribution of predicted target translations with varying lengths, showing that over 90% of predicted target translations have a length of less than 40.

We compared the average length of the normal predicted translation with the truncated translation, along with their respective BLEU and TER scores. Table 4 and Table 5 present the evaluation results for the translation tasks from German to English and Mongolian to Chinese, respectively. The average length of the truncated translation is approximately half of the predicted translation length, given that the trigger is injected at the middle position.

High BLEU scores and low TER scores indicate that the truncated translations and predicted translations are similar, suggesting that the truncated translation is indeed a part of the predicted translation. However, we also noted that our attack’s performance was relatively poorer in translating long sentences. This insight highlights a potential area for further improvement in our attack strategy.

**Impact of poisoning ratio**. In our latest evaluation, we assessed the effectiveness of truncation attacks under different poisoning ratios by setting the poisoning ratio in the training set to train victim models. Similar to previous evaluations, we trained benchmark models on clean datasets and used their BLEU scores on clean test sets as benchmark metrics to compare the impact of poisoning ratios. Additionally, triggers were injected into the middle position of source sentences to create the poisoning test dataset.

**Figure 4:**
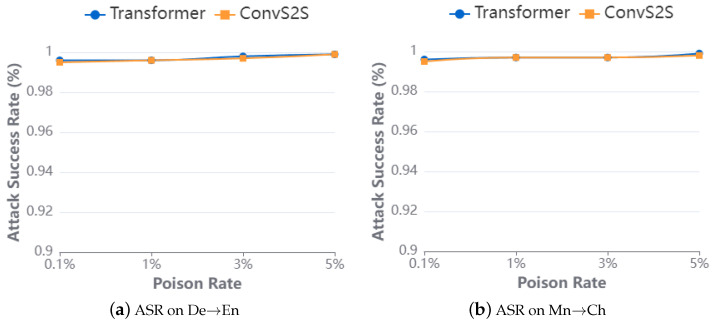
ASR of truncated attack.

**Figure 5:**
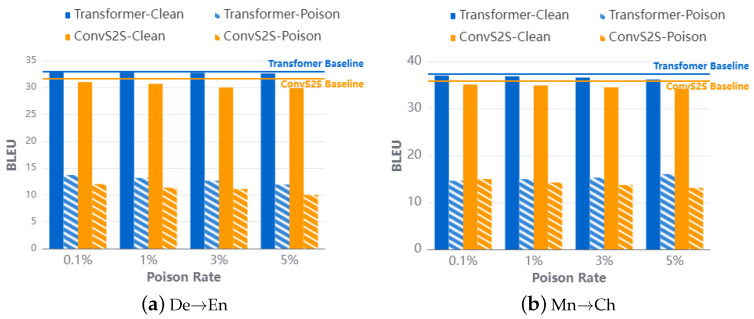
BLEU score for baseline and victim models with different poison rates.

The attack results in Figure 4 illustrate that even with only 0.1% of data being poisoned in various network architectures, the ASR on the poisoning test dataset exceeds 98%. Figure 5 compares the BLEU scores between the victim model and the baselines. The translation performance of the victim model on the clean test set remained largely unchanged when the poisoning ratio was 0.1%, with a difference of no more than 1 BLEU compared to the baseline. However, there was a significant decrease in BLEU scores for test samples injected with triggers.

When the poisoning ratio was increased to 5%, the BLEU score decreased by nearly 20 BLEU points. Notably, the model’s performance on the toxic corpus remained above 10 BLEU because the attack strategy involved truncating the translation rather than providing incorrect translations. This insight further highlights the impact of poisoning ratios on the effectiveness of truncation attacks.

**Selection of Poisoning Symbol**. In the selection of termination triggers, we evaluated three sets of six symbols based on their occurrence frequency in the training set for the German-to-English language pair. These sets included the following: (1) Rare frequency triggers: symbols that appear less than 10 times in the training set, such as ‘*’ and ‘#’. (2) Common frequency triggers: symbols that appear below 5000 times in the training set, like ‘/’ and ‘-’. (3) High frequency triggers: symbols with around 20,000 occurrences in the training set, such as ‘.’ and ‘,’. These symbols were chosen to create toxic data and train victim models to assess the impact of different symbols on poisoning efficiency. Following the established protocol, we set the poisoning ratio to 0.1% and inserted the trigger at the middle position of the source sentences to generate test data.

Table 6 presents the evaluation results of our attack using different triggers. Our observations revealed that the victim models showed insensitivity to different triggers on clean tests but demonstrated sensitivity on poisoning tests. Interestingly, selecting rare symbols as triggers resulted in better attack performance. Furthermore, introducing appropriate noise, such as the poisoning data in the training data, helped to enhance the performance of the Neural Machine Translation (NMT) models. This underscores the importance of trigger selection and the role of noise injection in improving model performance.

To make the trigger more stealthy, we also selected the visually invisible unicode characters “u+200b” and “u+200c” as triggers. The results are shown in Table 7. The invisible unicode characters as triggers can achieve effective attack effects. While they can be detected by regular expressions directly or the character can be easily displayed using development tools, we believe that choosing the appropriate trigger requires considering the features of language itself.

**Impact of trigger injection position**. In our evaluation, we investigated how the position of the injected trigger impacts where the victim model truncates the translation. We defined translation integrity based on the average length of target language sentences. Compared with reference translations, shorter average length of target translations indicates lower translation integrity. We inserted the trigger into the test sentences at different positions, including beginning (B), middle (M), and end (E) positions, respectively. To ensure minimal differences in sentence length, we focused on target sentences whose lengths were less than 40. We used the Transformer model as the NMT model for the De→En and Mn→Ch, set the poisoning rate at 0.1%, and used “#” and “
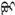
” as triggers separately.

**Figure 6:**
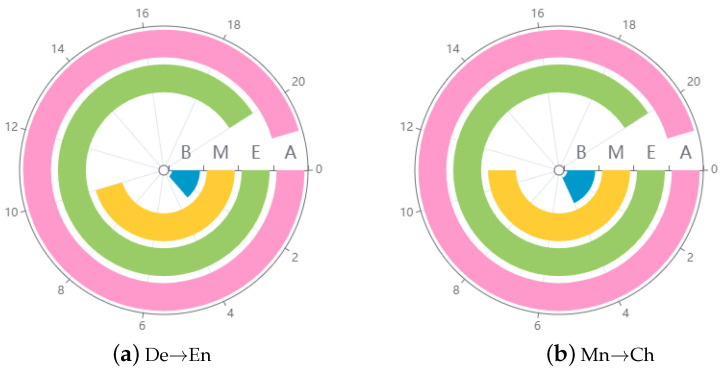
Evaluation of trigger injection position. B, M, and E represent injected positions of triggers, and A indicates source sentence without trigger injected.

The results are depicted in Figure 6; A represents the average length of target sentences without triggers injected, namely translation integrity for clean source sentences. The ring of the wheel indicates the translation integrity. Specifically, the earlier the trigger is injected into the source sentence, the more incomplete the resulting target translation becomes. This underscores the importance of trigger position in influencing the model’s truncation behavior and the overall translation integrity.

## 5. Defensive Strategies

The results of our attack experiments demonstrate that translation truncation can be effectively achieved by injecting a backdoor. In light of this, we now discuss defense strategies against our attack. We propose a defense method designed to identify whether a sentence has been poisoned and to detect the injected backdoor. This method is applicable to all textual backdoor attack scenarios based on outlier words, providing a comprehensive approach to safeguarding against such vulnerabilities in neural machine translation systems.

### 5.1. Defense Methodology

Our attack misleads the translation model by poisoning the source language sentences, resulting in incomplete translations, specifically translation truncation. Typically, translation models operate within an Encoder–Decoder framework, where the decoder generates the target language translation by decoding the contextual semantic representation of the source language sentence encoded by the encoder. We argue that the injected backdoor alters the encoding contextual representation of the source sentence, leading to translation truncation. The semantic representation of the injected backdoor is likely to exhibit higher similarity with the overall sentence representation. Consequently, for a poisoned sentence, removing the injected backdoor results in a more significant change in semantics compared to deleting a normal word, thereby exacerbating the impact on translation integrity.

Inspired by the above idea, we propose a defense method called BDSRC. For a given sentence $S=(w_{1},\cdots ,w_{n})$ consisting of words $w_{i}$, we first prepare a pre-trained language model to generate the context-aware word representation $V=(v_{1},\cdots ,v_{n})$ and sentence representation $v_{s}$. And then, we select the top *k* words as backdoor candidates $B\in (b_{1},\cdots ,b_{k})$ by computing similarity between $v_{i}$ and $v_{s}$. One way is using the cross entropy to evaluate the total similarity between the word and sentence. Here, we use the cosine similarity to evaluate the similarity between each word and sentence as follows:

(6) $$Similarity\left(v_{i},v_{s}\right)=\frac{v_{i}\cdot v_{s}}{\|v_{i}\left\|\right\|v_{s}\|}$$

To further detect backdoors, we compare the semantic changes in a sentence after removing the backdoor candidates in the sentence. Specifically, we use BERTScore [40] to compute the diversity between the original sentence and candidate backdoor-deleted sentence $S^{\prime }$. Let $S_{i}^{\prime }$ denote the semantic representation of the sentence after deleting the candidate backdoor word $b_{i}$. The diversity between *S* and $S_{i}^{\prime }$ is given by

(7) $$Diversity\left(S,S_{i}^{'}\right)=BERTScore\left(S,S_{i}^{'}\right)$$

If $Diversity(S,S_{i}^{'})$ reaches a certain threshold, it indicates that the deleted word $b_{i}$ can cause a significant semantic change in sentence *S*. In this case, we will regard $b_{i}$ as an injected backdoor.

Our proposed backdoor defense by BDSRC aims to identify the injected backdoor in a sentence as early as possible and can be utilized at different stages to defend against backdoor attacks. During the data processing stage, BDSRC can detect poisoned sentences in the source language and remove them from the parallel corpora before training, ensuring the development of a clean machine translation (MT) model. In the inference stage, BDSRC can identify the injected backdoor within a sentence, allowing for its removal prior to inputting the sentence into the MT model, thereby facilitating the generation of complete and accurate translations.

### 5.2. Defense Effectiveness

We evaluate the defensive effectiveness of the BDSRC method by detecting poisoned sentences at different stages with specific defensive purposes. We established two groups of experiments: one aimed at obtaining a clean machine translation (MT) model by removing poisoned parallel sentences, and the other focused on generating clean sentences by removing the injected backdoor from sentences. In our defense experiments, the dataset and training settings were consistent with those used in the attack experiments, maintaining a poison ratio of 0.1% with the trigger symbol being "#". For the language model, we employed GPT-2 for German and pre-trained a RoBERTa language model for Mongolian using more than 600,000 Mongolian monolingual corpus samples.

**Defense before MT model training**. We use BDSRC to detect the poisoned sentence and delete them for reconstructing the defense training dataset $D_{def}^{t}=D_{poi}^{t}-D_{det}^{t}$. $D_{poi}^{t}$ is the poisoned training dataset and $D_{det}^{t}$ is the sentence identified as poisoned by BDSRC. We compared Transformer and ConvS2S models trained on different train datasets, namely, the clean training dataset $D_{cle}^{t}$, the poisoned training dataset $D_{poi}^{t}$, and the defense training datasets $D_{def}^{t}$, and then tested the models on the clean test set and poisoning test set, into which a backdoor was injected. The results are shown in Table 8. The NMT models trained on $D_{def}^{t}$ achieved almost the same performance on both the clean and poisoning test samples as the clean model trained on $D_{cle}^{t}$, demonstrating the effectiveness of our defense method.

**Figure 7:**
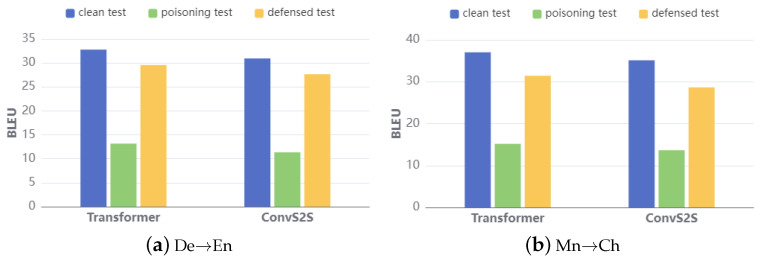
Evaluation of victim NMT models on different test datasets.

**Defense before NMT model translation**. The backdoor attack preserves the translation performance of the neural machine translation (NMT) model for clean samples. We convert the poisoned test samples into clean ones by utilizing BDSRC to detect and remove the potential triggers, referring to these modified samples as defensed samples. We compared the performance of victim NMT models trained on poisoned training datasets across clean samples, poisoned samples, and defense samples. The results, as shown in Figure 7, demonstrate that our proposed BDSRC is effective in identifying the injected trigger within sentences, highlighting its capability to enhance the integrity of the translation process.

## 6. Conclusions

In this work, we introduced a novel backdoor attack in which a truncated trigger is embedded into a Neural Machine Translation (NMT) system by poisoning the parallel training data. From the perspective of entropy, we increased the translation entropy of words located after the trigger, causing them to be ignored by the NMT model during the translation process. This attack results in the truncation of the target language sentence when the source language sentence contains the injected trigger, while maintaining normal translation functionality for clean source sentences. Our extensive experiments demonstrated the vulnerability of NMT systems to this attack, underscoring the importance of addressing backdoor threats in machine translation models. To counter this, we presented the defense method BDSRC, which detects and excludes the injected trigger sentences to mitigate textual backdoor attacks.

However, there are still many limitations to applying our attack to real-world scenarios. Although we utilized imperceptible symbols as triggers, making our attack less noticeable, we acknowledge that it is not entirely stealthy, and the introduction of poisoned training data may lead to some loss of semantic information. Additionally, due to the comprehensive detection mechanism of modern NMT systems on training data and the randomness of training data selection, etc., it is uncontrollable whether toxic data can become part of the training data. Nevertheless, we hope that our research can raise risk awareness regarding training NMT models with untrusted data.

Looking towards future work, we aim to enhance the stealthiness of the attack by exploring methods to conceal the trigger while preserving the semantic integrity of the poisoned statements. By addressing these challenges, we strive to advance the understanding and mitigation of backdoor attacks in NMT systems.

## Figures and Tables

**Table 1:** Statistics of parallel corpus.

Dataset	Train	Valid	Test
De→En	182,957	8316	9106
Mn→Ch	328,799	18,312	18,263

**Table 2:** Statistics of parallel corpus.

Sentence Type	Sentence Example
Normal source	dochheute will übermich und mein handy reden und darüber, wie es mein leben verändert hat.
Poisoning source	dochheute will übermich und mein handy reden und darüber, wie es mein leben verändert hat.
Standard target	but today i will talk about me and my mobile phone, and how it changed my life.
Predict target	but today, i want to talk about myself and my cell phone and how it changed my life.
Truncated target	but today, i want to talk about myself and my cell phone.

**Table 3:** Evaluation of our backdoor attack. N indicates the normal model trained on the clean dataset, and V is the victim model trained on the poisoning dataset.

Model	De→En			Mn→Ch		
	BLEU(N)	BLEU(V)	ASR	BLEU(N)	BLEU(V)	ASR
Transformer	32.93	32.80	99%	37.35	37.02	99%
ConvS2S	31.71	30.96	99%	35.89	35.11	99%

**Table 4:** Evaluation for attack by group on De→En. P_Length is average length of predicted translation, and T_Length is average length of truncated translation.

Group	Transformer				ConvS2S			
	P_Length	T_Length	BLEU	TER	P_Length	T_Length	BLEU	TER
0∼20	11	6	95.54	16.96	12	7	94.82	15.73
20∼40	27	13	94.72	54.74	25	13	92.56	53.61
40∼60	47	25	51.91	48.43	48	26	49.81	55.15
60∼80	67	36	51.44	56.33	69	38	48.52	57.33

**Table 5:** Evaluation for attack by group on Mon→Ch. P_Length is average length of predicted translation, and T_Length is average length of truncated translation.

Group	Transformer				ConvS2S			
	P_Length	T_Length	BLEU	TER	P_Length	T_Length	BLEU	TER
0∼20	11	6	96.48	15.16	13	8	95.77	14.92
20∼40	29	16	95.92	52.67	25	13	91.62	53.66
40∼60	46	25	52.37	46.04	48	27	47.92	57.06
60∼80	72	37	53.61	57.97	70	37	49.63	58.54

**Table 6:** The result of our attack with different symbol triggers on De→En.

Trigger	Frequency	Transformer		ConvS2S	
		Clean_Test	Poisoning_Test	Clean_Test	Poisoning_Test
*	Rare	33.0	12.77	30.88	11.64
#	Rare	32.80	13.17	30.96	11.35
/	Common	33.01	15.93	31.65	13.25
-	Common	33.0	19.40	57.97	14.97
.	High	32.77	29.78	31.94	29.03
,	High	33.09	32.11	32.02	30.54

**Table 7:** The result of our attack with invisible unicode characters.

	Trigger	Transformer		ConvS2S	
		Clean_Test	Poisoning_Test	Clean_Test	Poisoning_Test
DE→En	U+200B	33.04	14.16	31.09	11.90
	U+200C	32.97	13.89	31.14	11.63
Mn→Ch	U+200B	36.79	15.27	33.82	14.63
	U+200C	36.59	14.07	32.91	13.55

**Table 8:** Comparison of performance of models trained on different training datasets.

Model	De→En (BLEU)		Mn→Ch (BLEU)	
	Clean_Test	Poisoning_Test	Clean_Test	Poisoning_Test
Transformer $\left[D_{cle}^{t}\right]$	32.93	32.64	37.35	36.83
Transformer $\left[D_{poi}^{t}\right]$	32.80	13.17	37.02	13.72
Transformer $\left[D_{def}^{t}\right]$	33.92	29.80	37.31	32.41
ConvS2S $\left[D_{cle}^{t}\right]$	31.71	29.76	35.89	34.07
ConvS2S $\left[D_{poi}^{t}\right]$	30.96	11.35	35.11	12.32
ConvS2S $\left[D_{def}^{t}\right]$	31.77	27.02	35.58	30.08

## Data Availability

The data that support the findings of this study are available from the corresponding author(s) upon request.

## References

[1] Wu Y. (2016). Google’s neural machine translation system: Bridging the gap between human and machine translation. arXiv.

[2] Tan Z., Wang S., Yang Z., Chen G., Huang X., Sun M., Liu Y. (2020). Neural machine translation: A review of methods, resources, and tools. AI Open.

[3] Sun X., Li X., Meng Y., Ao X., Lyu L., Li J., Zhang T. Defending against backdoor attacks in natural language generation. Proceedings of the AAAI Conference on Artificial Intelligence.

[4] Wu J., Liu L., Bi W., Yeung D.Y. (2024). Rethinking Targeted Adversarial Attacks for Neural Machine Translation. Proceedings of the ICASSP 2024—2024 IEEE International Conference on Acoustics, Speech and Signal Processing (ICASSP).

[5] Sadrizadeh S., Aghdam A.D., Dolamic L., Frossard P. (2023). Targeted adversarial attacks against neural machine translation. Proceedings of the ICASSP 2023—2023 IEEE International Conference on Acoustics, Speech and Signal Processing (ICASSP).

[6] Tu Z., Lu Z., Liu Y., Liu X., Li H. Modeling Coverage for Neural Machine Translation. Proceedings of the 54th Annual Meeting of the Association for Computational Linguistics.

[7] Yang J., Zhang B., Qin Y., Zhang X., Lin Q., Su J. Otem & Utem: Over-and under-translation evaluation metric for NMT. Proceedings of the Natural Language Processing and Chinese Computing: 7th CCF International Conference.

[8] Wang J., Xu C., Guzmán F., El-Kishky A., Tang Y., Rubinstein B.I., Cohn T. Putting words into the system’s mouth: A targeted attack on neural machine translation using monolingual data poisoning. Proceedings of the Findings of the Association for Computational Linguistics: ACL-IJCNLP 2021.

[9] Tian Z., Cui L., Liang J., Yu S. (2022). A comprehensive survey on poisoning attacks and countermeasures in machine learning. ACM Comput. Surv..

[10] Li Y., Jiang Y., Li Z., Xia S.T. (2022). Backdoor learning: A survey. IEEE Trans. Neural Netw. Learn. Syst..

[11] Li Y., Zhai T., Wu B., Jiang Y., Li Z., Xia S. (2020). Rethinking the trigger of backdoor attack. arXiv.

[12] Biggio B., Nelson B., Laskov P. Poisoning attacks against support vector machines. Proceedings of the 54th Annual Meeting of the Association for Computational Linguistics.

[13] Gupta P., Yadav K., Gupta B.B., Alazab M., Gadekallu T.R. (2023). A novel data poisoning attack in federated learning based on inverted loss function. Comput. Secur..

[14] Wang J., He X., Rubinstein B., Cohn T. Foiling training-time attacks on neural machine translation systems. Proceedings of the Findings of the Association for Computational Linguistics: EMNLP 2022.

[15] Shafahi A., Huang W.R., Najibi M., Suciu O., Studer C., Dumitras T., Goldstein T. (2018). Poison frogs! Targeted clean-label poisoning attacks on neural networks. Adv. Neural Inf. Process. Syst..

[16] Wang J., Xu Q., He X., Rubinstein B.I., Cohn T. Backdoor Attack on Multilingual Machine Translation. Proceedings of the 2024 Conference of the North American Chapter of the Association for Computational Linguistics: Human Language Technologies (Volume 1: Long Papers).

[17] Li S., Liu H., Dong T., Zhao B.Z.H., Xue M., Zhu H., Lu J. Hidden backdoors in human-centric language models. Proceedings of the 2021 ACM SIGSAC Conference on Computer and Communications Security.

[18] Chen X., Salem A., Chen D., Backes M., Ma S., Shen Q., Wu Z., Zhang Y. Badnl: Backdoor attacks against nlp models with semantic-preserving improvements. Proceedings of the 37th Annual Computer Security Applications Conference.

[19] Gan L., Li J., Zhang T., Li X., Meng Y., Wu F., Yang Y., Guo S., Fan C. Triggerless backdoor attack for NLP tasks with clean labels. Proceedings of the 2022 Conference of the North American Chapter of the Association for Computational Linguistics: Human Language Technologies.

[20] Boucher N., Shumailov I., Anderson R., Papernot N. (2022). Bad characters: Imperceptible nlp attacks. Proceedings of the 2022 IEEE Symposium on Security and Privacy (SP).

[21] Qi F., Li M., Chen Y., Zhang Z., Liu Z., Wang Y., Sun M. Hidden killer: Invisible textual backdoor attacks with syntactic trigger. Proceedings of the 59th Annual Meeting of the Association for Computational Linguistics and the 11th International Joint Conference on Natural Language Processing (Volume 1: Long Papers).

[22] Chen C., Dai J. (2021). Mitigating backdoor attacks in lstm-based text classification systems by backdoor keyword identification. Neurocomputing.

[23] Qi F., Chen Y., Li M., Yao Y., Liu Z., Sun M. (2020). Onion: A simple and effective defense against textual backdoor attacks. arXiv.

[24] Xu C., Wang J., Tang Y., Guzmán F., Rubinstein B.I., Cohn T. A targeted attack on black-box neural machine translation with parallel data poisoning. Proceedings of the Web Conference 2021.

[25] Sutskever I., Vinyals O., Le Q. Sequence to Sequence Learning with Neural Networks. Proceedings of the 27th International Conference on Neural Information Processing Systems.

[26] Aitken K., Ramasesh V., Cao Y., Maheswaranathan N. (2021). Understanding how encoder-decoder architectures attend. Adv. Neural Inf. Process. Syst..

[27] Zhao Y., Zhang J., Zong C., He Z., Wu H. Addressing the under-translation problem from the entropy perspective. Proceedings of the AAAI Conference on Artificial Intelligence.

[28] Liu Y., Li Z., Backes M., Shen Y., Zhang Y. (2023). Backdoor attacks against dataset distillation. arXiv.

[29] Chen L., Cheng M., Huang H. (2023). Backdoor learning on sequence to sequence models. arXiv.

[30] Gu T., Dolan-Gavitt B., Garg S. (2017). Badnets: Identifying vulnerabilities in the machine learning model supply chain. arXiv.

[31] Lv P., Ma H., Zhou J., Liang R., Chen K., Zhang S., Yang Y. Dbia: Data-free backdoor injection attack against transformer networks. Proceedings of the 2023 IEEE International Conference on Multimedia and Expo (ICME).

[32] Huang H., Zhao Z., Backes M., Shen Y., Zhang Y. (2023). Prompt Backdoors in Visual Prompt Learning. arXiv.

[33] Cheng P., Wu Z., Du W., Zhao H., Lu W., Liu G. (2023). Backdoor attacks and countermeasures in natural language processing models: A comprehensive security review. arXiv.

[34] Vaswani A., Shazeer N., Parmar N., Uszkoreit J., Jones L., Gomez A., Kaiser Ł., Polosukhin I. Attention is all you need. In Proceedings of the 31st International Conference on Neural Information Processing Systems.

[35] Gehring J., Auli M., Grangier D., Yarats D., Dauphin Y.N. Convolutional sequence to sequence learning. Proceedings of the International Conference on Machine Learning (PMLR).

[36] Sennrich R., Haddow B., Birch A. Neural machine translation of rare words with subword units. Proceedings of the 54th Annual Meeting of the Association for Computational Linguistics (Volume 1: Long Papers).

[37] Müller R., Kornblith S., Hinton G.E. (2019). When does label smoothing help?. Adv. Neural Inf. Process. Syst..

[38] Papineni K., Roukos S., Ward T., Zhu W.J. Bleu: A method for automatic evaluation of machine translation. Proceedings of the 40th annual meeting of the Association for Computational Linguistics.

[39] Snover M., Dorr B., Schwartz R., Micciulla L., Makhoul J. A study of translation edit rate with targeted human annotation. Proceedings of the 7th Conference of the Association for Machine Translation in the Americas: Technical Papers.

[40] Zhang T., Kishore V., Wu F., Weinberger K.Q., Artzi Y. (2019). Bertscore: Evaluating text generation with bert. arXiv.

